# Professional and personal impact of the COVID-19 pandemic restrictions on non-COVID-19-related clinical research

**DOI:** 10.1017/cts.2021.16

**Published:** 2021-02-26

**Authors:** Ann J. Melvin, Pooja Tandon, Carly Rowe, Denae Clohessy, Tonya M. Palermo

**Affiliations:** 1 Department of Pediatrics, University of Washington, Seattle, WA, USA; 2 Institute for Translational Health Sciences, University of Washington, Seattle, WA, USA; 3 Seattle Children’s Research Institute, Seattle, WA, USA; 4 Department of Anesthesiology & Pain Medicine, University of Washington, Seattle, WA, USA

**Keywords:** Clinical research, COVID-19, impact

## Abstract

The COVID-19 pandemic has led to major disruptions in the clinical research enterprise. Investigators and staff at the University of Washington and Seattle Children’s Research Institute (SCRI) were surveyed to determine the impact of the pandemic restrictions on their non-COVID-19 clinical research studies. Enrollment and study visits were stopped for over half of the interventional trials. There were both positive and negative impacts of the work restrictions on the professional and personal lives of faculty and staff. Academic and research institutions should consider how best to support investigators and their teams during this pandemic.

## Introduction

Cases of SARS-CoV-2 infection first appeared in Washington state in January 2020 [1]. As the number of new diagnoses increased rapidly in Washington and around the country, there was a concern for overwhelming the healthcare system and steps were taken to contain the spread. On March 19, 2020, the University of Washington Medical Center (UWMC) and Seattle Children’s Research Institute (SCRI), an affiliate location, practice, and training site for the Department of Pediatrics at the University of Washington, issued orders for restricting human subjects research not related to COVID-19, mirroring the NIH guidance [2] issued on March 16, 2020.

Only visits that were critical for continued treatment or safety of participants or which could be done entirely in the context of a necessary clinical care visit or virtually could be conducted. Faculty and staff were required to work from home unless their need to be at work was mission-critical. As a result, non-COVID-19-related clinical research was significantly affected.

In this study, we aimed to assess the impact of the COVID-19 pandemic work restrictions on non-COVID-19-related adult and pediatric clinical research studies, as well challenges experienced by faculty and staff during this time that affected their ability to continue their research activities.

## Methods

An online survey administered via REDCap [3] was e-mailed to faculty, fellows, and research staff conducting clinical research at SCRI and UWMC on May 12, 2020 and July 6, 2020, respectively. The survey (available upon request) included multiple-choice and open-ended questions about the effect of the restrictions on currently active clinical research including interventional (i.e., studies evaluating the impact of treatment or preventive measures on the disease) and observational (i.e., studies observing the effect of risk factors without implementing a manipulation or intervention) studies, strategies employed and barriers to continuing research during the restrictions, the effect on study participants and professional and personal challenges encountered while continuing their clinical research activities. In order to protect participant privacy, the survey was anonymous and did not collect demographic information. This survey activity was considered as not human subject’s research by the University of Washington Institutional Review Board (IRB) review.

To prevent duplication of responses from multiple staff working on the same individual studies, only faculty responses were included for the questions specific to the number of clinical studies affected. Responses from all survey respondents were included for open-ended questions and those related to professional and personal challenges experienced while continuing their clinical research activities. Responses from fellows were grouped with those from research staff. Analysis was descriptive and included frequencies and percentages for categorical variables.

Open-ended responses on the survey were summarized using semantic thematic analysis. Two primary coders (TP and DC) familiarized themselves with the data by reviewing all responses to the open-ended survey questions. Codes were assigned by isolating the data into meaningful units of text that were semantically similar (i.e., conveyed the same sentiment even if using a different language). Next, the two coders worked together to group similar codes (e.g., IRB delay) into categories (e.g., organizational factors slowing research). The categories were then grouped into overarching themes (e.g., negative research impact).

## Results

Surveys were completed by 233 respondents (131 faculty, 91 staff, and 8 fellows, 3 missing category), with 51 responses from UWMC and 182 from SCRI. Overall response rate was 17%. Seventy-five faculty were leading at least 1 interventional trial and 78 at least 1 observational study. Forty-nine (32%) were leading both interventional and observational studies. Between them, the faculty were leading 200 interventional trials and 174 observational studies.

**Fig. 1.**
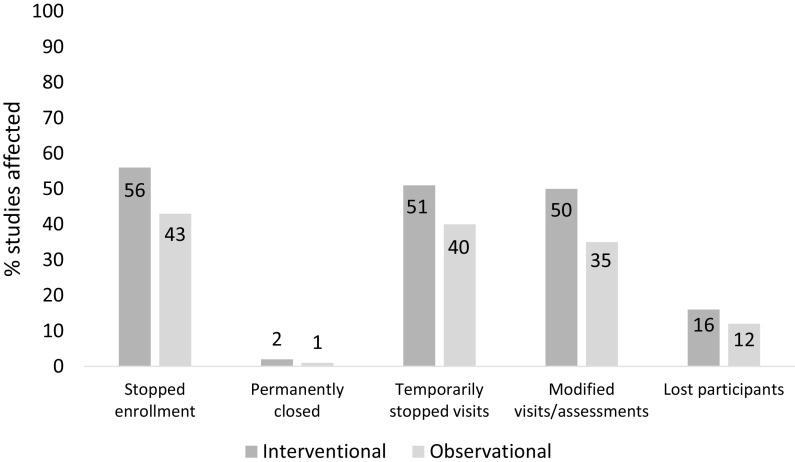
Effect of COVID-19 pandemic restrictions on studies conducted by faculty at the time of the survey. Interventional trials *n* = 200; observational studies *n* = 174.

Of the faculty leading interventional trials at the time of the shutdown, 57/75(77%) had to stop enrollment into at least one trial, with 34(45%) stopping enrollment into all their trials. Of the faculty leading observational studies, 43/78(55%) stopped enrollment into at least one study and 29(37%) stopped enrollment into over three quarters of their studies. Figure 1 shows how studies were affected by the restrictions.

Comments from respondents indicated that studies most affected were those requiring in-person contact for recruitment or study implementation. Studies less affected by the restrictions were those already using remote/virtual procedures, such as those utilizing online surveys or virtually delivered interventions.

Many investigators modified study procedures to be able to continue study activities during this time. The most common modifications to study procedures cited by faculty included switching to virtual study visits (50%) and obtaining consent remotely (41%). Other strategies used included widening study visit intervals (39%) and omitting or modifying some study procedures (36 and 33%, respectively). Fewer investigators used alternate venues such as the participant’s local clinic for study procedures (15%) or introduced or enhanced the use of various technologies such as mobile phone apps (16%).

Most investigators reported that they relied on their own study teams (>90%) to develop modifications to allow continued study activity and that they had the resources needed to be able to make the adaptations. Other sources of assistance included the IRB (21%) and other research teams (13%). Most investigators (76%) plan to incorporate at least some of the adaptations into future studies, particularly obtaining informed consent remotely and when feasible, virtual study visits. Several barriers to switching to remote study activities were cited by investigators. The IRB and research information technology services were prioritizing projects directly related to COVID-19, thus non-COVID-19 research activities received lower priority. There also were concerns about privacy from both the participant side (e.g., participants not wanting to give out their e-mail addresses) and from the staff side (e.g., staff now working from home and not wanting to use their personal cell phones to contact participants).

### Effect on Participant Experience

Few faculty (2%) reported safety/adverse events related to the change to more remote activities, although some voiced safety concerns about lack of ability to complete appropriate study procedures. A minority (23%) of the faculty noted it was harder to retain participants in their studies during this time due to stresses on participants and participant anxiety/reluctance to come to the hospital due to concern of exposure to COVID-19. A similar number (20%) thought it was easier to retain participants due to factors such as the flexibility of virtual visits and reduced travel and that more people were home and had time for study activities. When queried about factors that impaired or inhibited participants’ uptake of remote study activities, participant access to the necessary technology was reported most frequently as a challenge by faculty to whom the question was relevant (21/42).

### Personal Challenges

While faculty and staff reported similar challenges, the relative importance of each was different between faculty and staff (Table 1). There were some positive effects noted by the faculty including new collaborations (15%), increased time for writing and publishing (23%), and fewer meetings, events, and travel (40%). More staff than faculty reported concerns about returning to in-office/clinic work due to the lack of ability to maintain social distancing (46% faculty/64% staff), access to face masks (30% faculty/41% staff), and disinfectants/hand sanitizers (24% faculty/53% staff).

**Table 1.** Challenges experienced by faculty and staff related to the COVID-19 pandemic restrictions

Faculty		Staff	
Challenge	% affected	Challenge	% affected
Increased responsibilities at home	52	Barriers to technology	49
Concerns about grant funding and staff salaries	52	Inability to have what is needed at home*	33
Barriers related to technology	34	Worries about COVID-19 and personal safety	27
Increased clinical responsibilities	33	Increased responsibilities at home	25
Inadequate workspace	28	Inadequate workspace	4
Worries about COVID-19 and personal safety	23		
Inability to have what is needed at home*	16		

* Not able/allowed to bring study binder/regulatory binders home.

### Qualitative Coding of Open-ended Responses about Research and Personal Impact

Semantic analysis revealed several overarching themes about positive and negative research and personal impact, which align with quantitative results and provide additional context and details (Table 2).

**Table 2.** Qualitative coding of open-ended responses

Themes	Illustrative quotes
Positive research impact	
Remote procedures increased efficiency, ease of data collection, and responsiveness of participants	“If supported properly and if we can get youth/families equipped with devices, telehealth options and online surveys/econsenting are potentially much more convenient and feasible for ppts/families, especially those who live all over Washington state”
	“Remote consenting will broaden our reach in terms of recruiting diverse participants”
New research and grant opportunities related to COVID-19 research	“I pivoted to COVID-related basic science research”
	“opportunity to expand research to address COVID-19 impact”
Negative research impact	
Delays or stoppage of research	“2 studies basically placed on hold since they need in-person procedures”
	“I am unable to recruit to my study due to COVID. It’s unclear if funding will be extended or adequate when the time comes to resume recruitment”
Organizational factors impeding research progress	“If it’s possible to speed up IRB work, this has been an issue for several teams….it took over a month to hear back about a modification and another three weeks to have my response responded to”
Increased stress in families making certain studies or research procedures less attractive	“A few families were just too overwhelmed or stressed to do any study activities”
	“increased stress, caregiving demands, burden of ‘too much’ during COVID”
Not all study adaptations are working well	“Multi-site, longitudinal study - study visits have been delayed and broken up into online activities…- means double the scheduling/interactions since we need two ‘visits’ to get the info originally planned for one visit”
	“Patients had difficulty with device compliance remotely”
Positive personal impact	
Better work–life balance	“honestly a better work life balance - I can actually step away from work and workout then be back at my desk in a matter of steps. I am also able to care for an ailing pet who needed surgery without having to take time off work”
	“Not having to commute allows for more time for me to focus on my work and is less stressful”
	“work from home had proven to be very successful, I would like to continue a WFH status. It not only has reduced stress but has saved me money and allowed for more work hours”
Negative personal impact	
Work from home challenges due to technology, workspace, and lack of access to necessary equipment and supplies	“there are basic challenges and cost barriers in setting up a functional office at home - I have had to make purchases in order to be able to do my work at a similar level to that I would be doing in the office”
	“Lack of study supplies at home/need to send supplies to families/lack of adequate printer/scanner”
Work from home challenges due to child care/elder care responsibilities	“ ‘Increased time for writing and publishing’ can only apply to those who are going through this without small children”
	“ ‘Work from home’ with children has been a disaster and has led to the most stress and burnout I have encountered in my career thus far”
Mental health, worry about COVID-19, and return to work	“I worry about bringing COVID home to my family.”
	“Unpredictability about whether work arrangements will change again”
	“Isolation & Depression”
Worry about grant funding	“Uncertainty around future funding makes it hard to hire/retain staff”
Reduced collaborations and networking	“There has been less travel (zero travel) for academic meetings and events. That translates to less networking and reduced collaboration and generation of new ideas and projects”
Increased clinical workload	“Huge clinical admin burden completely wiped out my time for research”

WFH, work from home.

## Discussion

The COVID-19 pandemic has led to major disruptions in the clinical research enterprise. Our study demonstrated that during the early phase of the pandemic most investigators had to stop enrollment and study visits and modify study procedures for both their non-COVID-19-related interventional trials and observational studies at the time of the shutdown, consistent with other reports on clinical research from early in the pandemic. Most previous studies have reported on clinical trials only, finding that 20–87% of studies in various specialties at least temporarily halted enrollment [4–6]. Medidata Solutions, Inc. has been documenting the change in the activity of clinical trials, finding in April a 45% decrease in new patients entering study sites in the USA compared to the previous year [7]. While improved by August, new enrollments were still decreased by 22%. In April, two-thirds of investigators said they were halting or planning to halt patient recruitment in ongoing trials, half were delaying their new studies and over half were switching to virtual participant visits. These results were also somewhat improved by August [8].

Investigators responding to this survey were highly flexible in modifying trial procedures to continue clinical research despite the restrictions, some of which they intend to sustain. The most common modifications which many investigators plan to incorporate into future studies include conducting virtual study visits and using remote consenting. Fewer investigators introduced technologies that would allow remote clinical assessments, possibly due to lack of access to appropriate platforms or validated tools. Future work is needed to improve the ability to conduct study visits remotely including the development of tools to allow remote clinical assessments, creative trial designs to incorporate remote activities, and increased flexibility from IRBs and regulatory bodies to readily allow remote study activities.

The pandemic has also had a significant and far-reaching impact on the work–life balance of researchers and research teams, and their ability to continue their research activities. Some respondents did report positive impacts in terms of more time for activities such as writing. However, most faculty and many staff reported negative impacts including increased responsibilities at home. While we do not know the demographics of our respondents, the disproportionate challenges being faced by women [9,10] and people of color in medicine [11] and in academia during this pandemic have been highlighted, which could have repercussions for career advancement beyond the pandemic. A third of research faculty reported increased clinical workload, which could put them at risk for negative mental health consequences [12], in addition to a decreased capacity to continue their research activities. Worries about personal COVID-19 infection risk were also common, which could influence both individual well-being and workplace performance. Finally, the large proportion of faculty and staff with technology-related barriers to working from home is notable, in addition to not having adequate workspace and access to needed materials/supplies. It is likely that those technological challenges were magnified for some as the prolonged school closures required multiple family members to have individual devices, simultaneous internet access, and adequate workspaces.

It is important for academic and research institutions to consider how workplace and occupational interventions [13] can best support investigators and their teams during this pandemic and beyond. Our findings highlight organizational factors that make COVID-19-related research adaptations easier for research teams. Examples include simplifying the IRB approval and other processes related to virtually obtaining informed consent, adapting data collection or interventions to digital platforms, and distributing study incentives electronically to participants. In addition, research groups or institutes should foster the sharing of ideas and resources across research teams since we found that over 90% of teams developed their own modifications for pandemic circumstances. Online repositories of revised protocols or procedures developed by teams and online seminars to support the exchange of ideas would likely reduce redundant efforts and possibly increase collaboration. Other innovative strategies to support connection, productivity, and well-being should be explored, as have shown success in some academic settings [14] such as regularly scheduled virtual sessions to support and empower colleagues.

In addition, strategies to reduce barriers to research participation for those historically underrepresented in research and, now, experiencing health disparities related to COVID-19 are urgently needed. Organizations can explore opportunities for funding or creating shared access to devices (such as tablets, hotspots) that study participants can use for remote research procedures. They can support and encourage researchers to partner with communities to meet their needs and promote health equity during this challenging time.

To support the overall professional development and work–life balance of faculty and staff, organizations need to reduce barriers and inequities in effectively working from home. Workplaces should consider their critical role in supporting child care and elder care for employees, including innovative strategies such as helping facilitate “pods” (small groups of students that gather regularly to learn in a shared space) for families to share care responsibilities. In academic settings, promotions committees should consider more lenient policies related to tenure and promotion clocks and offer extensions and grace periods, as has been recently done at the University of Washington, recognizing the effects of COVID-19 on faculty productivity may extend for several years. Trainees and early stage investigators may be disproportionately affected by social isolation or additionally burdened by the care of young children during the pandemic. Options such as bridge funding, small internal grant opportunities, and institutional support for new grant applications could be effective strategies. Finally, many institutions have instituted protocols to safely allow access to office and lab spaces for essential research procedures during the pandemic. Similarly, when faculty and staff can safely return to work, institutions will need to clearly communicate protocols and policies for the business resumption and address the concerns of faculty and staff.

Our study has several limitations that should be considered. The overall response rate was low and may have been answered primarily by investigators who were more affected by the pandemic restrictions. We did not collect demographic data due to concerns about protecting the privacy of respondents within the institutions. We are not able to separately examine the impact by factors such as gender, age, race, family composition, or academic rank. Our data are limited to two institutions, and therefore may not be generalizable to other settings. The data were collected relatively early in the pandemic and the effect of the restrictions may have lessened over time. For example, our institutions have subsequently offered equipment to support working from home. However, as the pandemic has not yet abated, similar work and research restrictions remain in place. Further follow-up may identify additional impacts over the course of the pandemic.

In conclusion, our findings build on recent data demonstrating the impact of COVID-19 restrictions on clinical research and add the perspective of faculty and staff on both the professional and personal impact of COVID-19 restrictions.
